# Minimally Invasive Markers of Stress and Production Parameters in Dairy Cows before and after the Installation of a Voluntary Milking System

**DOI:** 10.3390/ani10040589

**Published:** 2020-03-31

**Authors:** Lucy J. Jerram, Steven Van Winden, Robert C. Fowkes

**Affiliations:** 1 Department of Pathobiology and Population Sciences, Royal Veterinary College, University of London, Hawkshead Lane, North Mymms, Hertfordshire AL9 7TA, UK; svwinden@rvc.ac.uk; 2 Endocrine Signaling Group, Department of Comparative Biomedical Sciences, Royal Veterinary College, University of London, Royal College Street, London NW1 0TU, UK; rfowkes@rvc.ac.uk

**Keywords:** dairy cattle, milking systems, hypothalamic-pituitary-adrenal axis, salivary cortisol, hair cortisol, lameness, milk yield, milk production

## Abstract

**Simple Summary:**

This research was carried out to investigate changing stress levels in dairy cows when changing from convention milking to an automatic milking system (AMS). Elevated stress levels can cause problems with immunity and reproduction. Stress in cows can be measured using cortisol levels found in saliva and hair (among others). AMS require fewer herds people and are associated with a higher milking frequency and higher milk yields. In this study, we present short-term and long-term cortisol levels in 10 and 12 cows respectively, over a period of 3–12 months. Hair and saliva samples were obtained with minimal restraint to the cow and analysed in the laboratory. Mixed models were applied to determine the effect of various parameters on cortisol levels. These show that non-lame cows had a reduction in salivary levels after AMS installation. Lameness and pregnancy affected salivary but not hair cortisol. Hair cortisol levels increased after installation, but this effect may have been seasonal. Milk yield increased and udder health improved across the whole herd but there was no change in the poor foot health. Our results show that AMS improves production and is not associated with an increase in cow stress. We recommend that welfare, natural behaviour and foot health are considered when introducing automatic milking systems.

**Abstract:**

Automatic milking systems (AMS) are a low-labour alternative to conventional parlours, with previous studies demonstrating that cows vary in their ability to cope with the change to AMS. Cortisol expression can be combined with other measures to assess stress: saliva and hair have the advantage of requiring minimally invasive sampling. No work has investigated the long-term impact of introduction of AMS. The aims of the study were to assess short-term and chronic stress associated with a change in milking system by measuring salivary and hair cortisol levels and to assess the impact on health and production parameters. Cows from one farm changing their milking system were recruited to the study and sampled for saliva (*n* = 10) and hair (*n* = 12) before and after installation. Cortisol levels were measured using a salivary cortisol enzyme immunoassay kit. Body condition, lameness and milk parameters of the whole herd were regularly assessed. Salivary cortisol showed no diurnal pattern but was affected by lameness and gestation. Non-lame cows showed a reduction in salivary cortisol after AMS introduction (*p* < 0.001). Hair cortisol levels increased after AMS, but it was unclear if this change was seasonal. Milk yield increased by 13% and somatic cell count reduced by 28%. Body condition score was consistently good, but lameness remained high throughout the study. Production values alone do not represent high welfare. The high lameness and associated cortisol levels suggest that cow stress requires consideration when changing milking systems.

## 1. Introduction

Cortisol (CORT) is the measurable end point of the hypothalamic-pituitary-adrenal (HPA) axis in all species and can be analysed in blood, saliva [1], milk [2], hair [3] and faeces [4]. The HPA axis is activated by stressful conditions, such as environment or management [5], and regulates reproduction and immune responses. Chronically high cortisol can have a detrimental effect on both the immune system and reproductive function [4]. Blood and salivary cortisol concentrations reflect short term HPA activity—typically minutes to hours—and can be affected by natural circadian rhythm or handling [6]. Faecal sampling removes any acute stressors and reflects cortisol concentration over the previous 24 h [4]. Hair cortisol measurements can also be used as a method for monitoring exposure to situations such as long-term chronic stress [7].

One advantage of assessing salivary, and particularly hair, cortisol concentration is that sampling is non-invasive. Salivary cortisol elevates twenty minutes after a stressful event, compared with blood which elevates after ten minutes [8]. This means that even if cows experience sampling-related stressors, it would not be reflected in the cortisol measurements. As hair samples represent middle-term trends in cortisol levels, neither circadian variations nor acute elevations in serum cortisol are reflected in hair cortisol concentrations [6,9].

Dairy cow hair grows at approximately 0.6–1 cm per month, with cattle undergoing a full moult every three months [10]. Cut hair samples of 2–3 cm, excluding the follicle, will therefore reflect average hormone levels and long-term cortisol variations. Hair cortisol concentrations appear to be affected by season, with contradictory studies showing higher levels in the winter [11] or summer [12]. Higher cortisol levels are associated with gestation and calving, [11], clinical disease [6,13] and multiple parities [3,13]. Long-term stressors need to be substantial—subclinical disease and chronic lameness do not increase hair cortisol concentrations [13,14]. Crossbreed cows have lower levels of cortisol (CORT) than Holstein Friesians, suggesting that hybrid vigour improves cows’ ability to cope with stressors [15]. At the lower end of the hair CORT range was a maximum concentration of 1.40 ± 1.08 pg/mg around calving [11], while another study reported mean concentrations of 11.7 pg/mg for cows in the control group [13]. A mid-point cut-off value of 4.17 pg/mg has been reported with a sensitivity of 62.4% and a specificity of 69.3%, separating ‘clinically compromised’ and healthy cows [15].

Salivary cortisol levels appear to mimic plasma cortisol concentrations but with a time lag [8] and yields around only 10% of plasma cortisol levels [16]. Due to cattle saliva collection issues [1], more work has been carried out on plasma which demonstrates a slight circadian rhythm peaking at around 6:00 am with a nadir around 6:00 pm and a distinct 120-min ultradian rhythm [17]. Salivary cortisol levels have been demonstrated to increase after adrenocorticotrophic hormone (ACTH) administration [1], as well as stressful events such as cow–calf separation [8], with a lesser increase after milking [1]. Normal salivary cortisol levels have been reported as between 0.02 and 0.06 µg/dL [8,18] while stressed cortisol levels were those between 0.13 and 0.65 µg/dL [8,19].

Conventional milking systems in the UK are varied and include rotary systems, herringbone parlours and tandem parlours. All conventional systems involve cows being milked either two or three times a day where they are held in a collecting yard before entering the milking parlour, many farms will have a backing gate to ensure a continued flow of cows through the system. Herds people are involved in preparing cows for milking, attaching milking machines and encouraging cows to enter the parlour. Automatic milking systems (AMS) may involve free, partially forced or fully forced cow traffic based on the dependence of passing through the AMS to access feed. Studies suggest that forced cow traffic improves milking frequency but reduces the expression of normal cow behaviour [20]. Previous studies have found no difference in cortisol levels between different AMS or tandem parlours [21,22], but other studies have shown higher cortisol levels in partially forced cow traffic AMS compared to herringbone or tandem parlours [23,24]. All cortisol levels in these studies were low compared to reported ‘stressed’ levels and it has been postulated that changes reflect the type of feed access more than the effect of different milking parlours. Regardless of parlour type, the longer the time spent milking, the more the cortisol level rises [21]—shorter but more frequent milking sessions may therefore reduce cortisol levels in dairy cows.

It appears that individual cows vary greatly in their ability to cope with AMS, with some adapting better than others. The ability to cope appears to be linked to waiting location rather than the parlour—waiting in collecting yards was linked to higher heartrates than in cubicles [25]. Overall, cows adapt quickly to new systems, as reflected by steady heartrates and low faecal cortisol metabolites [26].

No studies have assessed the long-term effects of cows changing from a conventional system onto an AMS. Therefore, the aims of the current study were, to determine diurnal changes in salivary cortisol levels in dairy cows, to assess short-term and chronic stress associated with a change in milking system by measuring salivary and hair CORT levels and to assess changes in health and production parameters associated with a change in milking system.

## 2. Materials and Methods

### 2.1. Selection of Cows

One commercial farm based in West Sussex, post code area GU29, was analysed in the study with all enrolled cows being part of the milking herd at this unit. 104 cows were in milk at the start of the study, with funds available for cortisol analysis of 13 cows. These were randomly selected from those deemed eligible using FarmWizard (FarmWizard Ltd., Belfast, UK), the herd management software, using a random number generator. Eligibility was based on the cows being present in the main herd from January 2018, with no mastitis or other chronic disease and no severe ‘score three’ lameness [27] at this time. The whole herd transferred from twice daily conventional milking (6:30 am and 3:00 pm) in a herringbone parlour to a free cow traffic AMS system using two Lely Astronaut A5 machines (Lely Holding S.a.rl., Maassluis, Netherlands) in February 2018, meaning no control group could be assessed. Cows in the herd ranged from first to eighth lactation, with the random selection used to produce a representative sample. Based on this selection, the sampling group consisted of cows from first to fifth lactation.

The milking herd were housed as one group in 120 cubicles throughout the year, with additional access to pasture in the summer months. Concentrates were fed at milking based on yield with ad libitum silage provided in feed troughs with open feed faces providing 82 cm per cow in the winter. In summer, cows were provided with ad libitum grass- and buffer-fed silage. Cubicles and feed areas were lit with low-level (30–50 lux) orange light from dusk to dawn. Cows are eligible for service (using Artificial Insemination (AI)) from 42 days post-calving, with pregnancy confirmed using rectal ultrasonography from thirty days after service. The average calving to conception interval at the start of the study was 88 days, with a calving index of 386 days. Cows spend 50 days dry between each lactation. Average lactation number was 2.77, there were 30 primiparous cows, 25 s lactation animals and 13 who were fifth parity (or higher) animals, and average daily yield was 33 kg/day. The herd was regularly observed by the farmer to monitor behaviour and disease, with a veterinarian called if intervention was required. A NACFT (National Association of Cattle Foot Trimmers) fully qualified foot trimmer visited every 6 weeks for both routine trims and lame cows.

Prior to commencement, the study was approved by the Clinical Research and Ethical Review Board at the Royal Veterinary College (approval date: 13 November 2017; URN: M2017 0112).

### 2.2. On-Farm Sampling

Whole saliva was collected using a plain Salivette tube (Sarstedt, Numbrecht, Germany), as described previously [28], six weeks before and six weeks after the introduction of AMS. Samples were taken at 6:00 am, 10:00 am, 2:30 pm and 6:00 pm for two days on both occasions. Sampling required cows to be restrained in an IAE Saracen cattle crush with a Superscoop^®^ addition (F. Klucznik & Son Ltd., Stoke-on-Trent, UK). All collection sessions lasted less than one hour, with cows remaining in the cubicle shed until required. One animal was too fractious at the first visit so was immediately excluded from the study. Following sample collection, saliva was stored at −80 °C until analysis.

Hair samples were taken 6 weeks before, 6 weeks after, 6 months after and 10 months after the introduction of AMS. Cows were restrained as for saliva sampling with the hair clipped from the inside of the right ear. This location was selected due to easy access and so as to not interfere with tuberculosistesting or any management procedures such as tail shaving. The milking herd were assessed for mobility and body condition score (BCS) [27,29] approximately every other month for the duration of the study by the main researcher to ensure consistency in recording. The ideal BCS is 2.5–3, with cows that are scored ≤2 defined as thin and cows that are ≥4 defined as fat [29]. Lame cows were any that scored 1, 2 or 3 on a scale of 0 to 3, where 0 represents a sound cow [27]. The temperature humidity index (THI) was calculated for a month prior to each hair sampling session using the equation: THI = ((1.8 × AT + 32) − (0.55 − 0.55 × RH) × ((1.8 × AT + 32) − 58) where AT is air temperature and RH is relative humidity [30]. Milk recording data was accessed from FarmWizard (FarmWizard Ltd., Belfast, UK) and the Lely AMS software (Lely Holding S.a.rl., Maassluis, Netherlands) to enable assessment of production parameters (average daily milk yield, daily milk fat percentage, daily milk protein percentage, somatic cell count (SCC), days in milk, frequency of milking and number of cows contributing to the bulk milk tank). No milk recording was carried out in the months immediately before AMS installation, so no data was available for this time period.

### 2.3. Laboratory Testing

Saliva and hair samples were measured for cortisol with a commercial, highly sensitive, salivary cortisol enzyme immunoassay kit (Salimetrics, Newmarket, UK; [28]). All saliva samples were assayed according to the manufacturer’s instructions (http://www.salimetrics.com/). Hair samples were finely cut into short sections to ensure efficient pulverisation and were weighed. The entire shaft of the hair samples was included in the analyses. Samples were powdered using a ball mill and a 5 mm stainless steel ball bearing at 30 Hz for 20 min. The powdered hair shafts were then immersed in 2 mL of absolute methanol for 18 h at 20 °C before centrifugation (3000 × G for 5 min at 4 °C). The supernatants were dried for a further 18 h at 60 °C before resuspension in the assay buffer supplied in the kit, and the absorbance measurement was set at 450 nm. The performance of the cortisol assay for hair linearity under dilution was assessed as described previously [31]. Salivary cortisol was expressed as micrograms of cortisol per decilitre and hair cortisol was normalised to picograms of cortisol per milligram. The inter- and intra-assay coefficients of variance were 11% and 3.4%, respectively.

### 2.4. Statistical Tests

Statistical analyses were carried out using IBM SPSS Statistics for Windows, version 26 (IBM Corp., Armonk, N.Y., USA). Paired T-tests and Pearson’s Correlation Coefficients were used to analyse individual measures. Data was checked for normality using the Kolmogorov–Smirnov test, if either the direct data, or the residuals of the (mixed) linear model, were not normally distributed, a log(10) conversion was applied. This was needed for the SCC. *p* ≤ 0.05 was considered statistically significant, with a post hoc power analysis confirming that the sample size for these parameters was adequate in all cases. The effect of sampling order was investigated by calculating Pearson’s Correlation Coefficients for each sampling session. There was no significant association between order of sampling and CORT concentration, so we did not further control for sampling order.

For the on-farm parameters, which were presented as averages or counts, a generalised linear modelling approach was taken, with a linear response or a Poisson distribution for counts (e.g., number of lame cows). Month was forced into the model to account for seasonal variation of the data. For salivary and hair cortisol analysis, mixed linear models were applied to identify any differences before and after the change from conventional milking to AMS. BCS was split into two categories (<3 and ≥3), and mobility was classified in a similar manner based on lame or non-lame cows. Stage of lactation, gestational status, lactation number, time of day and pre- or post-milking were also recorded for salivary cortisol analysis. Cow ID as a random variable and sample number were used as indicators to account for repeated measures using AR(1) (autoregressive process) as the autocorrelation structure. First, a univariate model was run identifying which independent factors were associated with CORT concentration.

## 3. Results

### 3.1. Production and Management Factors

**Figure 1:**
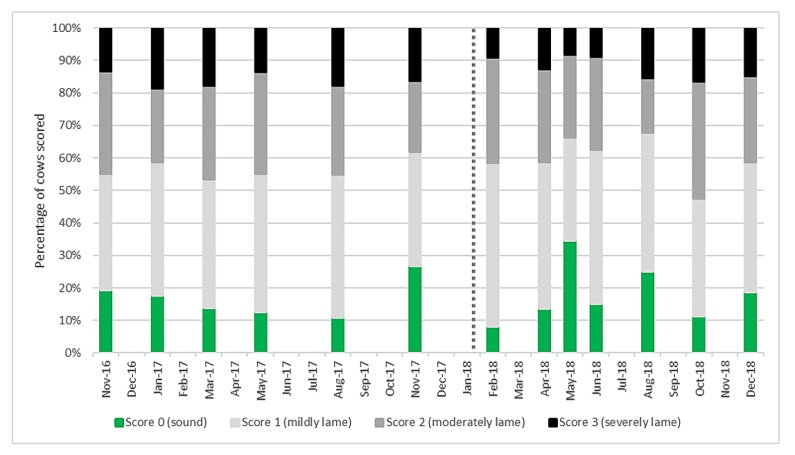
Mobility score results for the main milking herd (*n* = 101 ± 6) at each recording session from November 2016 to December 2018. Score 0 are sound cows, with scores of 1–3 representing worsening degrees of lameness. The vertical dotted line represents automatic milking system (AMS) installation.

Mobility scores both before and after the change to AMS (Figure 1) showed a high level of moderate and severe lameness compared to current UK levels of around 28% of the national herd (score 2 and 3) [32]. Lame cows (score 1–3) accounted for the majority of the herd at any recording session. The percentage of score 2 and 3 cows before AMS installation was 46% and after was 49% and there was no significant difference (*p* = 0.731).

**Figure 2:**
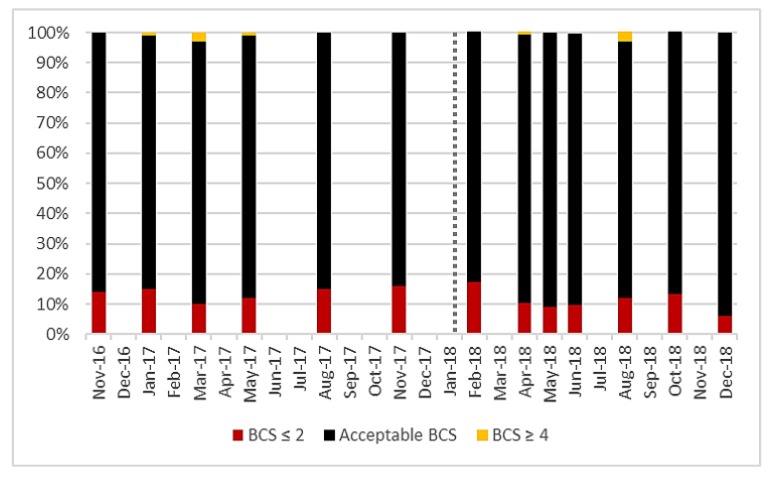
Prevalence of milking cows within the acceptable body condition score (BCS) range compared to over-fit or underweight cows observed at each BCS recording session for all lactating cows (101 ± 6) on the study farm from November 2016 to December 2018. Vertical dotted line represents AMS installation.

Acceptable body condition score values vary dependent on the stage of lactation, but most cows in the herd were within the prescribed range [29]. Before AMS installation, an average of 86% of cows were within the acceptable range and after, this was 89% (Figure 2), there was no significant difference (*p* = 0986). There was also no significant difference in the proportion of cows classified as thin before and after AMS installation (*p* = 0.580).

**Figure 3:**
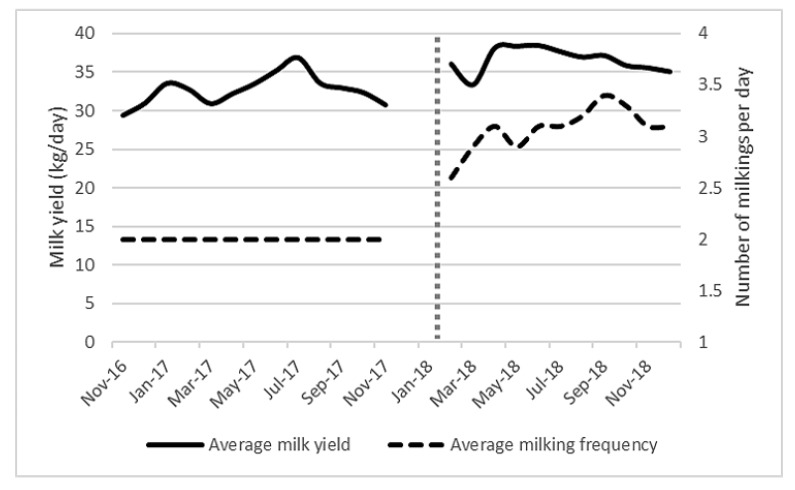
Average milk yield (kg/day) and average milking frequency (milking sessions/day) from November 2016 to December 2018 for all (101 ± 6) lactating cows on the study farm. Vertical dotted line represents AMS installation.

Figure 3 shows a significant increase in milk production (mean yield) after AMS installation (before = 33 kg/day, after = 36.5 kg/day, *p* = 0.008), which occurred without any significant difference in the average days in milk. The number of milking visits per day increased from 2 to 3.05, which was significant at the *p* < 0.001 level. It is difficult to determine whether the yield increase was due to the change in milking system or the increase in visits to the milking machine, as frequency of milking was highly correlated with both milk yield and month (both *p* < 0.001).

**Figure 4:**
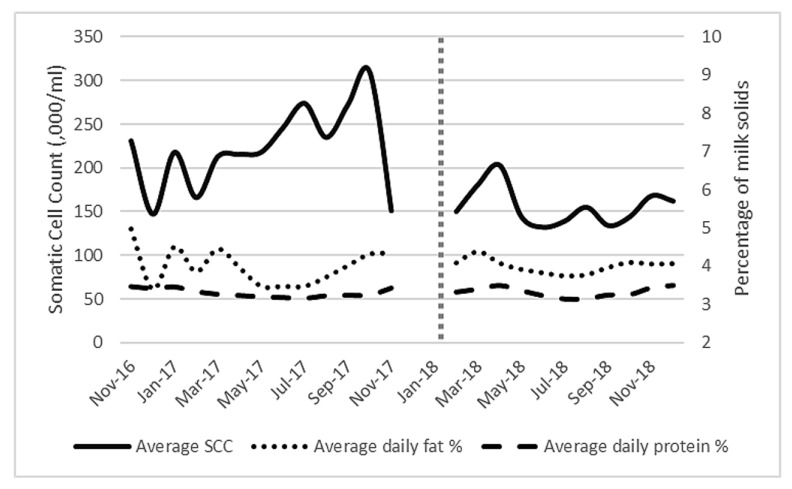
Average milk constituents (average daily fat and protein percentage) and average bulk milk somatic cell count (× 10^−3^/mL) for all lactating cows (101 ± 6) on the study farm from November 2016 to December 2018. Vertical dotted line represents AMS installation.

When milk constituents (Figure 4) were analysed, log(10) SCC was found to be significantly reduced after AMS installation (*p* = 0.001) to a level below the national average [33] (mean of 223,000/mL to 157,000/mL,). Fat percentage ranged from 3.39% to 4.96% while protein percentage ranged from 3.13% to 3.5% throughout the study. There was no significant difference in fat percentage (*p* = 0.325) or protein (*p* = 0.081) percentage before or after AMS installation. The multiple regression analysis identified that the main significant independent factor affecting milk production was frequency of milking (significant F change *p* < 0.001).

Significant correlations (Pearson’s coefficient) between production factors are summarised in Table 1, non-significant correlations have been excluded from this table. Average daily yield was positively correlated with average frequency of milking and month and it was negatively correlated with average somatic cell count and percentage of thin cows.

### 3.2. Effect of Introducing a Voluntary Milking System on Salivary Cortisol in Dairy Cows

**Figure 5:**
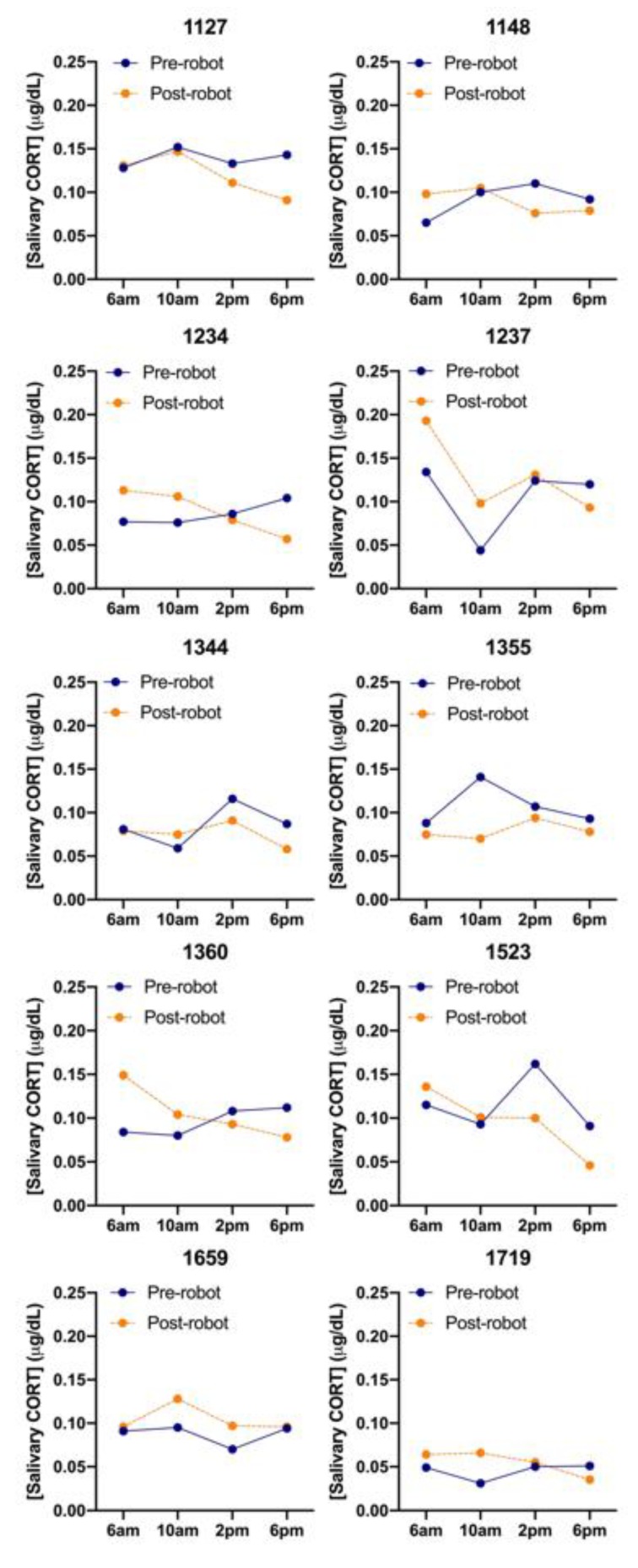
Salivary cortisol (CORT) levels (µg/dL) averaged for each sampling session (total samples = 160) for each individual cow (*n* = 10), before (solid line) and after (dashed line) AMS installation.

There was a wide variety in CORT levels across the ten cows that were sampled both before and after both morning and afternoon milking and in pre- and post-AMS installation (Figure 5). Cows #1237 and #1659 had higher CORT levels after AMS installation while #1344 and #1355 had lower CORT levels. The first 6:00 am pre-installation sampling session had significantly lower average CORT readings than all the following sessions (*p* < 0.05), except for the first 10:00 am pre-installation and final 6:00 pm post-installation sampling session. There was no relation with saliva cortisol level with BCS (*p* = 0.179) or time of day (*p* = 0.386), but cows tended to have 0.0118 (±0.0064) µg/dL higher saliva cortisol level before milking (*p* = 0.068). Cows that were pregnant had a 0.0156 (±0.0067) µg/dL higher level than non-pregnant cows (*p* = 0.021), the number of days in milk was negatively, but not significantly, associated with salivary cortisol (*p* = 0.080).

As presented earlier, the herd was experiencing more lameness after the AMS installation. In order to account for the potentially stressful event of said lameness, we evaluated the saliva cortisol levels of lame (score 1 and over) and non-lame cows in before and after AMS conditions, presented in Table 2.

The results show that sound cows have lower levels of cortisol after the introduction of the AMS, whilst lame cows were not affected (*p* = 0.118). Lame cows in post-AMS conditions have higher levels of saliva cortisol, whilst the reverse is the case for pre-AMS conditions.

### 3.3. Effect of Introducing a Voluntary Milking System on Hair Cortisol in Dairy Cows

**Figure 6:**
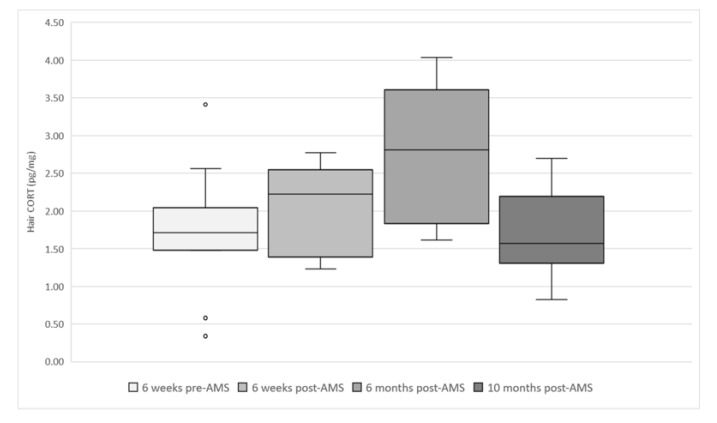
Box and whisker plots showing hair cortisol levels (pg/mg) at each sampling session (total samples = 47) in evaluated cows (*n* = 12) from six weeks before AMS installation (January 2018) to 10 months (November 2018) after AMS installation.

Figure 6 shows the long-term cortisol levels in the hair of the evaluated cows. The readings over time were significantly different (*p* = 0.027). The post hoc test revealed that 6 months post-AMS installation was 0.9826 (±0.3393) pg/mg higher than the reading pre-AMS (*p* = 0.038) and 1.0114 (±0.3595) pg/mg higher than 10 months post-AMS (*p* = 0.047). The THI range was 52–75 for the month prior to the 6 months post-AMS sampling session—all other maximum THIs were less than 58. No other parameters (lameness, BCS, pregnancy, stage of lactation) were significantly associated with the level of cortisol in the hair.

## 4. Discussion

This study demonstrates the importance of using multiple parameters to assess cow adaptations to a new milking system. Across all cows, neither short- nor long-term cortisol levels showed any significant difference between milking systems. However, considering the non-lame cows, there is a significant lowering of the saliva cortisol levels, suggesting the adaptation is favourable short term. The main changes to production factors were an increased frequency of daily visits to the milking machine, increased milk yield and reduced somatic cell count. For all factors, a control group would have ensured that the significant results identified were due to the tested variables. All cows on the farm transitioned to the AMS at the same time, preventing comparisons between the new and old system. Comparing the study cows with cows from a separate conventional milking farm would have resulted in many confounding factors around farm size, location, cow genetics and handling stress, among others. Therefore, the best option was to compare the cows to themselves throughout the course of the study.

### 4.1. Salivary Cortisol Analyses

Previous research suggested that the baseline level for salivary CORT was around 0.048 µg/dL, with values over 0.16 µg/dL associated with stress [8,34,35]. The range in this study was from 0.0075 to 0.2676 µg/dL despite no notably stressful events occurring. This difference in measurements is likely to be due to the use of different assay kits. When comparing calf research that used the same assay kit, the unstressed levels had a wider range while stressed levels exceeded 0.1416–0.234 µg/dL [18,19] (Supplementary Figure S1). Only seven of all salivary CORT values (*n* = 160) were above 0.16 µg/dL, with five of these being associated with samples taken before milking. This is confirmed by the mixed model showing a tendency towards lower CORT levels at the after-milking sampling sessions (0.0118).

Negrao et al. argued that saliva sampling should not be considered as a stress-free sampling method in adult cows as it took them six times as long to obtain an adequate saliva sample compared with a blood sample [1]. Creation of a stress-free sampling method with no necessary human interaction would be ideal. In this study, we only restrained the cows for an average of 90 s to obtain the samples, this was possible due to the use of a ‘super scoop’ function (F. Klucznik & Son Ltd., Stoke-on-Trent, UK) which reduced the amount of time needed for each cow.

Sampling order had no effect on salivary CORT levels, which showed that the study cows were not anticipating restraint during the sampling sessions. This, combined with previous research showing that cows do not intrinsically find being in a cattle handling system stressful [35], suggests that our salivary CORT data do reflect the true level in these animals without being affected by the sampling process. When lameness was accounted for, non-lame cows had lower cortisol levels after AMS installation, which suggests they had coped acutely well with the changes in milking system.

It was surprising that lame cows had lower CORT levels before AMS installation despite being under chronic stress. Long-term lameness may have reduced the cows’ ability to respond to acute stressors due to reduced pituitary responsiveness, meaning their salivary CORT remained low [36]. Score 1 cows contributed to the higher salivary cortisol levels after AMS installation, which suggests that they are still responding to the acute stress of novel lameness. Very few thin cows were assessed, therefore it was not possible to investigate the effect of negative energy balance on salivary CORT levels. Cows with a higher body condition had higher CORT levels, which is likely also related to their metabolism and risks of fatty liver [37] as well as physical size constraints associated with milking stall or parlour width. AMS was not associated with any change in salivary CORT levels and samples taken after milking were consistently lower, suggesting that time spent in the milking stall did not induce any short-term cortisol increase. Unlike plasma cortisol levels, no diurnal changes were observed [17].

Hair cortisol levels have previously been found to increase throughout pregnancy [11] but no research has shown later gestation to be associated with increased salivary cortisol levels. No cows were over 24 weeks pregnant in this study, but we still identified that pregnant cows had a higher salivary cortisol level than non-pregnant cows, suggesting that saliva mimics hair cortisol levels despite generally being associated with acute stressors.

### 4.2. Hair Cortisol Analyses

The mean hair cortisol concentration in this study was 1.99 ± 0.77 pg/mg, which is similar to the levels reported previously and lower than the cut-off value of 4.17 pg/mg separating healthy and sick cows [6,14]. Hair CORT after AMS installation was significantly higher, particularly associated with the August sampling session. Hair CORT has been shown to vary throughout the year. One study of Holstein cows found that June was associated with the highest CORT levels (13.0 ± 1.0 pg/mg, n = 18) [38], while another assessing Swiss dairy breeds identified winter as the season with the highest CORT levels (0.86 ± 0.37 pg/mg, *n* = 27) [11] (see Supplementary Figure S2 for comparisons). Our data agreed with the former rather than the latter, which may be linked to the maximum THI for July reaching 75. A THI ≥ 74 is recognised as the upper critical THI for dairy cattle [30], so there was at least one day in the month before sampling that would have been classified as causing heat stress to the study cows. Without multiple years being analysed, it is hard to confirm which factor caused the increase in hair CORT in the August session. If this change truly is seasonal, then this increase may not be associated with AMS and could be linked to heat stress, increased walking or changes to feeding method [30,32,38].

Seventeen samples (*n* = 47) were taken from cows showing signs of lameness in the month before the session. Previous research showed no significant difference in hair CORT between lame and non-lame cows with a similar sample size [14] and our results reflect this, therefore cows showing lameness were retained in the study. Multiparous animals did not have significantly different CORT to primiparous animals in this study, which contradicts previous results [7]. The cows sampled all had ‘non-stressed’ cortisol levels throughout the study, which may be why no difference was observed. Behaviour was not analysed but at sampling, the two youngest cows were the easiest to sample and had the shortest flight distance.

The ear was a convenient sampling site when the cows were in the crush for saliva sampling but required more restraint than many other locations. One cow (#1719) allowed the hair to be cut with no restraint of any kind but all the other cows required the same restraint as for saliva sampling. All hair used in this study was black, which has been shown to have lower CORT concentrations than white hair [3]. Sampling hair from the tail switch would have required less restraint while growing quickly and is white in Holstein cattle [7]. However, farmers often trim the tail switch as a management tool which could affect sampling; additionally, the risk of environmental or faecal contamination would have been high.

### 4.3. Milk Production Parameters

It was anticipated that the high level of lameness would reduce during the study period, but this was not the case, with a slight but non-significant increase observed. After AMS-installation, the cows had less time standing before milking and no need to turn sharp corners into and out of the parlour, which should have reduced the incidence of sole ulcers and white line disease, respectively [39,40]. The type of lameness was not quantitively assessed—other causes include sole bruising (which can worsen to an ulcer) and digital dermatitis. Cows were foot-bathed straight out of the old parlour five times a week with a 5% formalin footbath, which is commonly used by farmers to treat treponeme-related digital dermatitis [41]. Due to the new system having two AMS machines and a lack of space at the exit to the rear machine, there were practical issues preventing regular foot bathing. The foot trimmer recorded higher levels of digital dermatitis later in 2018 associated with winter housing. Initially, the farmer reported a high number of cows kicking the AMS units, which was not associated with an immediate increase in lameness. The cows were most lame in the summer when their walking distances increased with turn out. This highlights the importance of considering all management-related issues before a change in milking system. The farmer’s main aim during 2019 was to improve the regular provision of an appropriate foot bath and better manage the cow tracks. This should help to reduce the lameness prevalence to a more acceptable level, which in turn should increase the number of visits made to the AMS [42]. It may be that the cows that adapted best to the new milking system were the ones with more foot problems and thus they have been retained when they may have otherwise been allocated as cull cows.

Our body condition score data reflects previous work that showed no difference in body condition score with the change to AMS [43,44]. This suggests that the cows on this farm have adapted well to a decreased interval between milking sessions without any adverse effect on energy balance. The study farm chose to have free cow traffic, which ensures there is continuous access to cubicles and feed troughs. Natural behaviour can be expressed with no impact on feeding or ruminating behaviour, which can inhibited with forced cow traffic [45,46].

Frequency of milking and month (pre/post-AMS) of the study were highly correlated, making it difficult to determine whether the almost 13% yield increase was due to the change in milking system or the increase in milking visits. Milking frequencies of 1.4 to 3.2 times per day have been reported, with the study farm more closely matching the higher end [47,48,49]. Early lactation animals and those with a lower parity have been shown to visit the AMS the most [21,50]. There was no significant difference in the average days in milk across the duration of the study, so this was not a factor in milking frequency. Additionally, this suggests that milk yield was not impacted by a sudden increase in freshly calved cattle. We found no difference between fat and protein content, which were comparable to recognised UK figures [33] and reflect individual cow variation. Somatic cell count decreased by over 25% during the study, likely linked to improvements in teat preparation and better consistency in milking routine. Farms that move to AMS without considering important factors, such as manure management, udder hygiene or the risks of over-reliance on automated systems, are more likely to have increases in SCC [51,52]. AMS can have a positive effect on udder health and sub-clinical mastitis [53,54,55] and the machines’ ability to detect early changes in udder health will only continue to improve with further research by the manufacturers.

## 5. Conclusions

On this farm, there was no overall difference in salivary cortisol levels associated with a change in milking system, but when only sound cows were assessed, there was a significant reduction in salivary cortisol levels after AMS installation. Cows did not appear to have consistent diurnal changes in salivary cortisol. Instead, timing of milking sessions had more of an effect on short-term cortisol levels. After AMS installation, hair cortisol levels increased, peaking in August. More work needs to be carried out assessing the long-term impact on AMS transition to determine whether this increase in cortisol was affected by seasonality or other confounding factors. Milk yield increased by 13% after the installation of AMS, with a reduction in SCC of 28% and no negative impact on milk constituents. Body condition remained good, suggesting that feed intakes and ruminating times were not adversely affected by the change in system. Production values alone do not equate to high welfare and the high levels of lameness on the farm combined with its effect on salivary cortisol suggest that cow stress continues to need consideration when changing systems on commercial dairy farms.

## Figures and Tables

**Table 1:** Significant correlations ^1^ between production parameters for all lactating cows (101 ± 6) on the study farm from November 2016 to December 2018.

Production Parameter	Average Daily Yield	Average Milking Frequency	Month	Average Days in Milk	Number of Cows in Milk
Average daily yield		0.753	0.693		
Average milking frequency			0.882		
Average days in milk					−0.572
Average SCC	−0.366 (*p* = 0.033)	−0.625	−0.486 (*p* = 0.006)		
Lame cow percentage				0.370 (*p* = 0.031)	−0.456 (*p* = 0.01)
Thin cow percentage	−0.331 (*p* = 0.049)		−0.344 (*p* = 0.042)		

^1^*p* < 0.001 unless otherwise stated. SCC: somatic cell count.

**Table 2:** Saliva cortisol levels (µg/dL) and standard deviations in sound and lame cows in both pre- and post-AMS conditions.

Saliva Cortisol (µg/dL)	Sound Cows (Score 0)	Lame Cows (Score ≥ 1)	Significance Level
Pre-AMS	0.12704 (0.05549)	0.08807 (0.03834)	*p* < 0.001
Post-AMS	0.05499 (0.01916)	0.09863 (0.03616)	*p* = 0.003
Significance level	*p* < 0.001	*p* = 0.118	*p* < 0.001

## Supplementary Materials

The following are available online at https://www.mdpi.com/2076-2615/10/4/589/s1, Figure S1: Salivary cortisol reference ranges for ‘unstressed’ and ‘stressed’ cattle based on previous studies adjusted to µg/dL where necessary [8,18,19,33,34], Figure S2: Hair cortisol levels (pg/mg) by season comparing previous studies with the current work [11,37]. Table S1: Jerram et al. 2020 original data.
