# Supplementary figures and images for: Minimally Invasive Markers of Stress and Production Parameters in Dairy Cows before and after the Installation of a Voluntary Milking System
Source: Animals (Basel). 2020 Mar 31;10(4):589. doi: 10.3390/ani10040589 (PMC7222793; doi:10.3390/ani10040589)

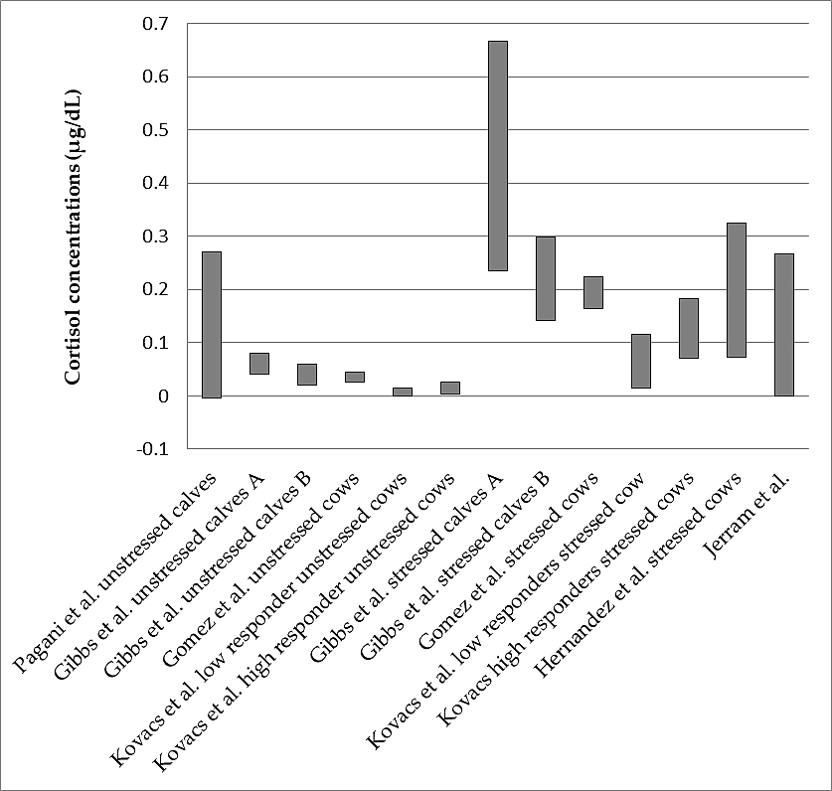

Supplement: Supplementary file 1 [file animals-10-00589-s001.zip › Figure S1. Salivary cortisol reference ranges for unstressed and stressed cattle.tif]

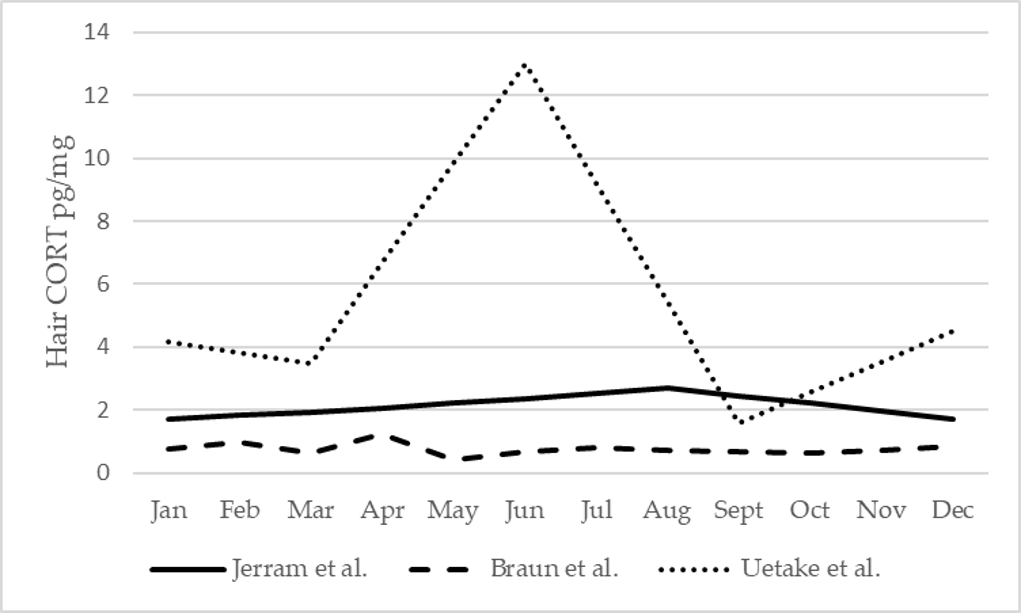

Supplement: Supplementary file 1 [file animals-10-00589-s001.zip › Figure S2. Hair cortisol levels by season comparing previous studies.tif]
